# The potential of component-resolved diagnosis in laboratory diagnostics of allergy

**DOI:** 10.11613/BM.2018.020501

**Published:** 2018-04-15

**Authors:** Slavica Dodig, Ivana Čepelak

**Affiliations:** Department of medical biochemistry and hematology, Faculty of Pharmacy and Biochemistry, University of Zagreb

**Keywords:** allergy, IgE, component-resolved diagnosis

## Abstract

The initial laboratory approach in the diagnosis of allergies is to detect the type of allergic reaction, *i.e.* whether the patient’s allergy is mediated by immunoglobulin E (IgE) or not. For this purpose, the concentration of total serum IgE (tIgE) and specific IgE (sIgE) are determined. Progress in laboratory diagnostics is the use of component-resolved diagnosis (CRD) which implies determination of sIgE against purified native and recombinant allergenic molecules. Component-resolved diagnosis is used in laboratory practice as singleplex and multiplex assays. The choice of allergen for singleplex assay is based on anamnesis, clinical findings of a patient and on skin prick test results. Multiplex-microarray assays simultaneously determine multiple sIgE’s against numerous allergens. The goal of CRD is to distinguish the true allergens from the cross-reactive allergen molecules. Component-resolved diagnosis allows predicting the risk of severe symptoms, as well as anticipating the development of allergies. Thus, determination of sIgE against allergenic components may significantly improve current diagnostics of allergy. Since this method is applied in laboratory practice just a few years, it is necessary to acquire new knowledge and experience, to establish good co-operation between specialist in medical biochemistry and laboratory medicine and the specialist allergologist, so that the method can be applied in a rational manner. Component-resolved diagnosis will significantly improve the diagnostics of IgE-mediated allergy in the future. The aim of this article is to present potentials of CRD in the laboratory diagnostics of allergy mediated by IgE.

## Introduction

The initial laboratory approach in the diagnosis of allergies (such as atopic eczema, food allergy, rhinitis and wheezing disorders) is to detect the type of allergic reaction, *i.e.* whether the patient’s allergy is mediated by immunoglobulin E (IgE) or not. For this purpose, the concentration of serum total IgE (tIgE) is determined. Today, the determination of tIgE concentration, as a simple and automated method, is an integral part of the screening process for subjects with atopy (*1*). Thereafter follows the procedure for identification of allergens which triggered allergic reaction, by determination of specific IgE (sIgE) against possible causative allergens to which the skin test, history and clinical picture of the patient were pointed out (*2*, *3*). Determination of sIgE concentration over a number of years implied identification of sIgE by allergenic extract materials derived from natural allergen source materials. Progress in laboratory diagnostics of IgE-mediated allergy is the use of component-resolved diagnosis (CRD) or molecular diagnosis of allergies. Component-resolved diagnosis implies determination of sIgE concentration against purified native and recombinant allergenic molecules (*4*-*6*). Natural allergenic molecules may be purified by chemical, chromatographic, electrophoretic and/or immunoaffinity techniques from allergen extracts of natural allergen source materials. Production of a recombinant allergen is a highly complex process comprising a whole series of procedures including extraction and isolation of messenger RNA (mRNA) from allergenic source, complementary DNA (cDNA) synthesis, electrophoretic separation of each component of the allergen source, primer preparation for polymerase chain reaction (PCR), multiplication of cDNAs of individual allergenic components and finally expression of recombinant allergens (*e.g. rBet v1, rBet v2, rBet v4, etc.*) in appropriate systems, most commonly in bacterium *Echerichia coli* (*7*).

Component-resolved diagnosis, which is based on the determination of sIgE concentration against individual allergenic molecules, allows detection of sensitisation against individual components of the allergic source, even against those lacking in the allergen extract (*6*, *8*). The development of DNA technology has enabled the introduction of individual allergenic molecules into laboratory diagnostics of allergies. Since the CRD has been applied in recent years, future investigation will examine the diagnostic power of this currently expensive method. The aim of this article is to present current potentials of CRD in the laboratory diagnostics of allergies.

## Allergens

Allergens are substances that, in hypersensitive subjects, with a predisposition to enhanced IgE synthesis, may stimulate immediate-type hypersensitivity reactions mediated by IgE (*i.e*. the type I hypersensitivity reaction) (*9*). The reaction takes place in two phases: a) the phase of sensitization against the causal allergen (IgE antibodies bind to high-affinity IgE receptors (FcεRI) on the surface of mast cells and basophil granulocytes), and b) repeated exposure to the same allergen, leading to cross-linking of IgE on sensitized cells, and the consequent release of mediators of allergic reaction from sensitized cells.

Genuine allergenic molecules imply major allergens, which in most patients cause a primary, species-specific sensitization (induce synthesis of sIgE) and consequent allergic reaction. Cross-reactive allergenic molecules, due to their similarity to major/genuine molecule, can only cause allergic reaction after previous contact with the main sensitizer, and they induce mild allergic reactions (*6*). Cross-reactivity occurs when the similarity with the species-specific molecule is greater than 70%, but it is rare if the similarity is less than 50%. At the same time, the major epitope should be located at the molecular surface accessible to its IgE antibody (*10*).

Immunoglobulin E can bind to a cross-reactive molecule within a similar type of allergen sources (*e.g.* within mites or within grasses) and can also bind to stable molecules with similar functions in the various types of allergenic components belonging to the same protein family (*e.g*. within profilins, tropomyosins, lipocalins, calcium binding proteins, *etc*.) (*6*). It also enables detection of crossreactive carbohydrate determinants (CCDs), which have no clinical relevance (*11*).

Allergens can enter the organism mostly by inhalation, ingestion or after skin contact (*12*-*22*).

In everyday practice, the notion of an allergen is often not used precisely - sometimes this term is used to designate an allergenic source, sometimes to designate an allergenic protein, and in the last few years, this term is understood to mean an allergenic molecule. For a better understanding, this can be substantiated by an example: allergenic sources (house dust mite) and single allergenic protein/molecules (*Dermatohagoides pteronyssinus* proteins *Der p1, Der p2, Der p3, etc.*). It is also important to define the following terms: major, minor, primary, and cross-reactive allergenic molecules, respectively. Generally, major allergenic molecule may bind to IgE in > 50% of allergic patients with an allergy to its source react, and minor allergenic molecule may induce allergic reaction in < 50% of clinically allergic patients with an allergy to its source react (*5*, *6*). Genuine allergenic molecule is a molecule that causes specific sensitization to its corresponding allergen source. A primary allergenic molecule is the driving trigger, *i.e.* the original senziting molecule in a particular patient. Major allergenic molecules can be defined more precisely as primary or genuine allergenic molecules. In addition to these terms, it is important to understand the phenomenon of cross-reactivity, which implies IgE’s ability to bind to allergenic molecules (homologues) other than the target allergenic molecules present in different species and then induce an immune response. Therefore, due to their shared, similar or identical epitopes, cross-reactive molecules (homologues) may react with IgE in the same way as target allergens. Cross-reactivity will occur if the similarity of the primary structure with the target allergene molecule is greater than 50 - 70% (*6*). In addition, cross-reactivity will appear if there is a reaction between IgE and CCDs (*23*-*25*). For each patient it is important to detect sensitization to genuine allergenic molecules and to detect cross-reactive molecules. Co-sensitization implies simultaneous hypersensitivity to allergenic molecules from different allergenic sources (*e.g.* weeds and birch) - this sensitization does not result in cross-reactivity. Attempts to define an allergen always fall into the definition of a function, according to which allergens, originating from plants, animals and microorganisms, could be defined as those antigens that are capable to stimulate the type I hypersensitivity reactions in hypersensitive persons. Allergens can be classified into several groups, such as inhalant, nutritional, contact, hymenoptera venom allergens, *etc*. (Table 1).

**Table 1:** Classification of allergens according to sources

**Allergens**	**Source**	**Characteristics**	**Reference**
Inhalant	grass, weed, tree, mites, *etc*.	Hypersensitivity to inhalant allergens can result in the appearance of allergic rhinitis, asthma or conjunctivitis. Diagnostic sensitivity and specificity of sIgE against alergenic extracts are different depending on the type of allergenic extract. The existence of cross reactivity within certain groups of allergens has been established, as well as between inhalant and nutritive allergens (*e.g.* fruits, vegetables, nuts, *etc.*)	6,12-16
Nutritive	fruits, vegetables, nuts, seeds, beans, nuts, *etc*.	Food sIgE is sensitive for detecting of food-allergen sensitization, but clinical specificity is limited. Recently, CRD, being rapidly incorporated into laboratory diagnostics, enables distinguishing the genuine from cross-reactive allergens. Moreover, it has better diagnostic specificity than sIgE against allergenic extracts.	6,17-19
Contact	latex, *etc.*	Clinical symptoms include contact dermatitis, allergic contact dermatitis (hypersensitivity type IV), and urticaria, angioedema, rhinitis, conjunctivitis, bronchospasm, and anaphylaxis (type I hypersensitivity reaction). sIgE to at least 10 allergenic molecules may be identified.	6,20-22
Hymenoptera venom	Honey bee, wasp, *etc*.	Persons allergic to stinging insect venom are at risk for a much more serious allergic reaction, *i.e.* anaphylaxis. False positive results of sIgE are possible due to carbohydrate moieties of glycoproteins.	6,23-25
sIgE – specific immunoglobulin E. CRD – component-resolved diagnosis.			

Currently allergens could be defined as proteins, glycoproteins, lipoproteins, or protein-conjugated haptens, which have unique molecular and structural properties [*e.g.* relative molecular mass (Mr) 5 to 150, isoelectric point (pI) 4 to 7, carbohydrate composition, nucleotide and/or amino acid sequence] (*26*). Allergenic molecules belong to different protein/peptide groups with different functions in the pathomechanism of allergic reactions, resulting in a well known and previously mentioned functional allergen definition (*26*). The main allergenic sources are foods, fungi, trees, weeds, grasses, mites, and finally animals; with the largest number of allergenic proteins being found in foods and the smallest in animals (*27*). Individual allergenic proteins/peptides belong to the following protein groups/families: storage proteins (11S globulins, 2S albumins i 7S vicilins), nonspecific lipid transfer proteins (nsLTP), pathogen-related-10-proteins (PR-10-P), profilins, lipid binding proteins (LBP), lipocalins, calcium-binding proteins, enzymes, defensin-like proteins, serum albumin, tropomyosins, heat-shock proteins (*28*-*45*).

Each of the above mentioned proteins consists of a larger or smaller number of molecules that have stronger or less pronounced allergenicity, *i.e.* can be defined as genuine or cross-reactive allergenic molecules (*6*). So far, a number of molecules of individual allergenic proteins have been isolated and produced, for example: birch allergen *Bet v* (*Betula verrucosa*) contains 8 molecules, mite allergen *Der p* contains 23 molecules, peanut allergen *Ara h* (*Arachis hypogaea*) contains 17 molecules, honey bee allergen *Api m* (*Apis mellifera*) contains 12 molecules, wasp allergen *Ves v* (*Vespula vulgaris*) contains 6 molecules, *etc.* (*6*). New molecules of allergenic proteins and peptides are discovered every day. Contemporary laboratory allergy diagnostics allows identification and quantification of exactly these individual molecules.

## Allergen molecules as members of protein families

**Figure 1:**
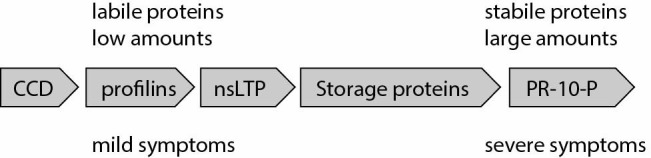
Increasing risk for manifestation of severe symptoms depending on allergenic protein type. CCD - cross-reactive carbohydrate determinants. nsLTP - nonspecific lipid transport proteins. PR-10-P - pathogen-related-10-proteins.

According to the function of some allergenic molecules (Table 2) it may be possible to predict the severity of the patient’s symptoms (*6*). Hypersensitivity to labile proteins (*e.g.* CCDs, profilins) can cause local, milder symptoms, and hypersensitivity to stable proteins (*e.g.* storage proteins, PR-10-P) may imply a risk for systemic, more severe symptoms (Figure 1).

**Table 2:** Function of some proteins and allergenic molecules

**Protein**	**Function**	**Allergenic source**	Allergenic molecules*
Storage proteins (11S globulin, 2S albumin i 7S vicilin)	biological storages of ions and amino acids	plant seeds, nuts, milk, egg whites	*Cor a9, 14 Ara h1,2,3,4,6* *Ber e1, Jug r1, Ses i1*
nsLTP	transfer of phospholipids	fruits, trees, weeds, cereals, nuts	*Mal d3, Pru p 3, Cor a 8, Jug r3, Pla a3 Amb a6, Art v3, Zea m14, Gly m1, Cas s1, Ara h9, 16, 17*
PR-10-P	defense against microbes or insects, chemicals	plants	*Bet v1; Ara h8; Mal d1, Cas s1*
profilins	actin binding proteins	trees, weeds, fruits	*Bet v2, Phl p12, Art v4, Ole, Cit s2, Cuc m2 Mus a1, Mal d4, Ara h5, Gly m3*
LBP	lipid binding proteins	mite, cockroach, cat, dog, plants	*Der p2,7,13, Der f7*
lipocalins	transport of hydrophobic molecules (steroids, retinol, lipids); PG synthesis	cattle, dog, cat horse	*Bos d2,5, Can f1,2, Fel d4, Equ c1*
calcium-binding proteins enzymes	transfer of calcium	trees, grass, weeds, fish	*Bet v4; Phl p7*
isoflavone reductase	biosynthesis of isoflavonoid phytoalexin	plants	*Bet v6, Cor a6, Ole e12*
peptidil prolyl isomerase	interconverts cis/trans isomers of peptide bonds with the amino acid proline	trees	*Bet v7*
glutathione S-transferase	catalyze the conjugation of reduced form of glutathione to xenobiotic substrates		*Der p8, Der f8*
cysteine protease	catabolism and protein processing	mites	*Der p1, Der f1*
alpha-amylase	hydrolyses α-bonds of large, α-linked polysaccharides	mites	*Der p4*
pectate lyase	eliminative cleavage of pectate	weeds	*Art v6*
defensin-like protein	antimicrobial peptides, acts as disruptors of microbial membranes	weeds, nuts	*Amb a4, Art v1, Gly m2, Ara h12,13*
serum albumin	carrier protein	animals	*Can f3, Fel d2*
tropomyosins	maintenance of cell morphology	pan-allergen - foods, dust mite, cockroach	*Der p10, Bla g1*
heat-shock proteins	response to exposure to stressful conditions; antigen presentation	mold, chestnut	*Alt a, Cas s9*
*name of allergenic molecules according to the latin name of allergenic source (http://www.allergen.org). nsLTP - nonspecific lipid transfer proteins. PR-10-P - pathogen-related-10-proteins. LBP - lipid binding proteins. PG - prostaglandin.			

## Application of single allergen molecules

For many years, *in vivo* and *in vitro* allergy diagnostic procedures have applied allergenic extracts, *i.e.* mixtures of allergenic and nonallergenic molecules. Purification, production and research of individual allergenic molecules dates back to 30 years ago when the *Der p1* of house dust mite *Dermatophagoides pteronyssinus* was cloned in *Echerichia coli*, resulting in the production of rabbit antiserum anti-*Der p1*, followed by cloning the cockroach allergen (antigen 5, now known as *Dol m5*), and the main birch allergenic molecule *Bet v1* (*46*-*48*). Since then, large numbers of purified native allergenic molecules and recombinant allergenic molecules have been isolated and produced (inhalant and nutritive allergens, allergens from insect venom, mold and latex).

The structural characteristics and functions of recombinant allergen molecules correspond to the characteristics of natural allergen molecules. Equal quality of recombinant molecules in all series of preparations and the fact that they are not subject to either genetic or biological variations, ensures excellent reproducibility of the tests in which they are applied (*6*). Scientific research with individual molecules includes examination of allergic reaction pathomechanisms, application in skin prick tests (SPT), possible application in allergen-specific immunotherapy (ASIT), and *ex vivo* methods, which investigate the activation of basophilic granulocytes with individual allergic molecules, *i.e.* basophil activating test (BAT) (*49*-*51*). Allergen-specific immunotherapy would be indicated if oligo/mono sensitization to the genuine allergenic molecules is confirmed. Preparations for component-resolved immunotherapy (CRI) may be based on the mixtures of allergenic determinants derived from one source. These preparations are designed as hypoallergenic derivatives (*52*). Numerous studies have focused on the testing of allergenic activity of various preparations, *e.g.* recombinant allergenic mosaics (containing ≥ 2 proteins), fragments, oligomers and chimeras/hybrids. To be effective, CRI preparations as well as SPT preparations should preserve both, allergenic activity and ability of induction of pro-inflammatory cytokines. Therefore, conformational-dependent B-cell epitopes need to be removed, and T-cell epitopes to be preserved. For the present, these properties have not been achieved for most CRI preparations (*52*). When these problems would be solved satisfactorily, individual molecule preparations will be able to be applied for SPT and ASIT. For now, single native and recombinant allergens are used in laboratory diagnostics of allergy as CRD.

## Methods used in component-resolved diagnostics

Component-resolved diagnostics is used in laboratory practice in two main types of assays, *i.e.* as singleplex and multiplex assays. Singleplex assay implies the determination of one single allergen (one sample, one allergen) (*53*).

The assay is based on standardized sandwich fluoro-immunochemical or lumino-immunochemical method on a three-dimensional carrier. Allergen component, covalently coupled to carrier, reacts with the specific IgE in the serum sample. After washing away of non-specific IgE, enzyme-labelled antibodies against IgE are added, forming a complex. Then, incubation and washing away of unbound enzyme-labeled anti-IgE are followed. The bound complex is then incubated with a developing agent. After stopping the reaction, sIgE is determined by a fluorometric or luminometric analyzer (*54*, *55*). Multiplex assay is also based on fluoro-immunoassay. This method simultaneous determines the concentration of sIgE’s to a broad spectrum of allergen components. The difference exists in the immunoassay carrier and the detection method: allergen molecules (components) are immobilized on a solid substrate in a microarray format, and final image acquisition using an appropriate microarray scanner. The results obtained must be analyzed with proprietary software (*56*).

The choice of allergen for singleplex assay is based on anamnesis, clinical findings of a patient and on SPT. Multiplex-microarray assays simultaneously determine multiple sIgE’s against numerous allergens, *e.g*. sIgE’s to 120 allergens. In addition to allergen sensitization profiles, a list of clinically insignificant molecules is also obtained (*57*). The results are expressed semi-quantitatively in ISU-units, *i.e.* ISAC Standardized Units for specific IgE (Table 3) according to the recommendations of the test manufacturer, which are standardized according to the World Health Organization IgE standard (WHO 75/502).

**Table 3:** Interpretation of serum concentration of sIgE

**ISU**	**Interpretation of serum concentration of sIgE**
< 0.3	undetectable or very low (0)
0.3 – 0.9	low (*1*)
1 – 14.9	moderate to high (*2*)
≥ 15	very high (*3*)
ISU – ISAC Standardized Units. sIgE – specific IgE.	

Diagnostic accuracy of CRD varies depending on the type of allergens, and diagnostic sensitivity is greater than the diagnostic specificity. For nutrititive allergens the sensitivity is in the range of 66 - 100%, and the specificity ranges from of 0 - 95% (food chalenge tests were used as a gold standard) (*58*). Diagnostic accuracy of CRD for inhalant allergens is difficult to determine because there is no gold standard but CRD results can be compared with the method for determining sIgE using allergenic extracts or with SPT results.

## Benefits of the use of CRD

Component-resolved diagnosis has a significant contribution to the diagnosis of allergy as well as in therapy. The main goal is to distinguish the true allergens from the cross-reactive allergen molecules. Therefore this method should be applied in the following cases: a) in patients with anaphylaxis caused by various cofactors (*e.g.* effort, non-steroidal anti-inflammatory drugs), in patients with delayed anaphylaxis (3 - 6h) after consummation of red meat, or in patients with idiopathic anaphylaxis; b) in individuals with latex allergy; c) in subjects with multiple hypersensitivity, *i.e.* sensitivity to pollen and plant food allergens; d) in subjects with food allergy - to predict the severity of symptoms, depending on the type of protein, and to identificate allergens that could eventually lead to allergic symptoms (increased sIgE concentration - currently no symptoms; sIgE are detectable in the serum even years before symptoms); e) to facilitate the choice of genuine allergen for ASIT (in individuals with hymenoptera venom allergy, in patients with pollen polysensitization and in subjects with inhalant oligo/monosensitization) (*8*, *59*).

Given the different allergenicity of certain types of protein molecules, CRD allows predicting the risk of severe symptoms, as well as anticipating the development of allergies. Thus, determination of sIgE against allergenic components significantly improved the diagnosis of allergy. Also, it can be expected that CDR will improve *in vivo* diagnostics in the future - scientists expect the SPT to be replaced with CDR (*60*). Besides, as the CRD enables identification of genuine and cross-reactive components, this method will help in the individualized approach of ASIT, since ASIT should be applied if the patient demonstrates hypersensitivity to genuine allergens (*5*, *61*, *62*).

## Necessity for careful interpretation of CRD results

Critical approach to the application of this method implies a good co-operation between the specialist in medical biochemistry and laboratory medicine and the specialist allergologist, and the common consistency in the interpretation of the obtained results (*63*, *64*). The final interpretation is influenced by the pre-analytical, analytical and post-analytical phase of the CRD. In the pre-analytic phase, it is necessary to be aware that expensive multiplex assay is not indicated according to clinical symptoms of allergy. Therefore, sometimes redundant results may be obtained. It is also important to recall that the concentration of sIgE varies with seasonal exposure to allergens so that the measurement of sIgE concentration makes sense within two to six months after exposure to a causal allergen (*65*). The fact that the sensitivity or specificity of CRD is not the same for all allergic molecules, refers to the analytical phase. For the correct interpretation (post-analytical phase) it is particularly important to note that increased concentration of a single molecule reveals hypersensitivity but does not have to mean that the molecule is the cause of the symptoms (*66*). The latter may lead to unnecessary food elimination in case if all LTP will be eliminated from the diet. The results of a single patient should not be projected to the whole population, as CRD is foreseen for individual diagnostics. Currently, CRD can not replace double-blind placebo-controlled food challenge test in case of peanut allergy (*67*).

In conclusion, CRD is a great challenge for specialists in medical biochemistry and laboratory medicine and for specialist allergologist. The advantage of CRD over the current methods for determining the concentration of sIgE is that CRD can distinguish the true allergen molecules from the cross-reactive allergen molecules; CRD enables the detection of the risk of severe symptoms and predicting the development of allergies for each individual patient. In that way the CRD provides a possibility for a personalized approach to the patient with allergy.

Laboratory reports require an interpretative commentary from a specialist in medical biochemistry and laboratory medicine to facilitate the clinical interpretation of numerous data in the laboratory report. Continuous education of specialist in medical biochemistry and laboratory medicine and expert allergologist is needed, so that this expensive method can be applied in a rational manner. In the hands of an experienced allergologist, CRD findings can significantly contribute to individualized approach to the patient. Otherwise, this method may be unnecessary use of expensive reagents/tests.
