# Corrigendum to: The potential of component-resolved diagnosis in laboratory diagnostics of allergy

**DOI:** 10.11613/BM.2018.021201

**Published:** 2018-06-15

**Authors:** Slavica Dodig, Ivana Čepelak

**Affiliations:** Department of medical biochemistry and hematology, Faculty of Pharmacy and Biochemistry, University of Zagreb

This is a correction of *Biochemia Medica* 2018;28(2):020501. DOI: https://doi.org/10.11613/BM.2018.020501

Since the publication of this article, the authors have noticed that the Figure 1 was published incorrectly. The correct Figure 1 is presented below. The authors apologize for any inconvenience caused to the readers.

**Figure 1:**
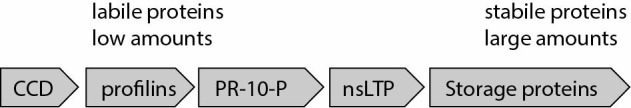
Increasing of the risk for manifestation of severe symptoms (6). CCD - cross-reactive carbohydrate determinants. nsLTP - nonspecific lipid transport proteins. PR-10-P - pathogen-related-10-proteins.

